# Cigarette smoking and cardiovascular disease incidence and all-cause mortality: the modifying role of diet quality

**DOI:** 10.1186/s12889-024-18468-z

**Published:** 2024-04-12

**Authors:** Mostafa Norouzzadeh, Farshad Teymoori, Hossein Farhadnejad, Nazanin Moslehi, Parvin Mirmiran, Seyedeh Tayebeh Rahideh, Fereidoun Azizi

**Affiliations:** 1 Department of Nutrition, School of Public Health, Iran University of Medical Sciences, Tehran, Iran; 2 Nutrition and Endocrine Research Center, Research Institute for Endocrine Sciences, Shahid Beheshti University of Medical Sciences, Tehran, Iran; 3 Endocrine Research Center, Research Institute for Endocrine Sciences, Shahid Beheshti University of Medical Sciences, Tehran, Iran

**Keywords:** Cardiovascular diseases, Mortality, Mediterranean diet, Smoking, Adults

## Abstract

**Background:**

This study examines the potential long-term joint association between smoking and diet quality as modifiable risk factors concerning cardiovascular diseases (CVDs) incidence and all-cause mortality among current and former smokers.

**Methods:**

The study followed 955 smokers from the third and fourth examinations of the Tehran Lipid and Glucose Study to March 2018. Dietary data was collected using a food frequency questionnaire. Three diet quality indices (DQIs) were computed at baseline: DQI-international (DQI-I), DQI-revised (DQI-R), and Mediterranean-DQI (Med-DQI). Cox proportional hazards regression models were used to determine the HR (95% CI) of the joint association between smoking and diet quality among heavy and light smokers, based on the number of cigarettes per day and pack-years, as well as between current and former smokers based on smoking habits.

**Results:**

Over a follow-up period of almost eight years, 94 cases of CVDs (9.80%) and 40 cases of mortality (4.20%) were documented. The lower diet quality based on the Med-DQI was associated with a higher risk of mortality among current smokers (HR:3.45; 95%CI:1.12, 10.57). Light smokers with good diet quality, compared to heavy smokers with poor diet quality, had a lower risk of CVDs incident (HR:0.35; 95%CI: 0.15, 0.83) and all-cause mortality (HR:0.20; 95%CI:0.05, 0.77). Current smokers with good DQI had a lower risk of mortality compared to current smokers with poor DQI (HR:0.26; 95%CI:0.08, 0.80). However, this lower risk was more significant in former smokers with good DQI (HR:0.10; 95%CI:0.02, 0.45).

**Conclusions:**

Light and former smokers had a lower risk of developing CVDs and experiencing mortality. However, when coupled with a high-quality diet, this protective effect is even more pronounced.

**Supplementary Information:**

The online version contains supplementary material available at 10.1186/s12889-024-18468-z.

## Background

Cardiovascular diseases (CVDs) are currently the primary cause of death worldwide [1]. Meanwhile, Iran is also experiencing an upward trend in CVDs incidence [2]. CVDs are multifactorial conditions that result from the interaction of genetic, metabolic, and environmental factors [3, 4]. Smoking and poor diet are among the modifiable and greatest contributors to the global burden of CVDs [5]. The total number of smokers has increased globally, leading to almost 8 million deaths and 200 million disability-adjusted life years (DALYs) in 2019 [6]. Notably, poor diet is responsible for 10.9 million deaths, and 255 million DALYs annually [7].

Smoking and poor diet share common disease-causing mechanisms that can interact and increase the risk of CVDs over time [8]. Smokers often adopt unhealthy dietary habits, which are characterized by lower intake of fiber and higher consumption of fats and sugar [9, 10]. Empirical evidence suggests that smokers with inadequate nutritional status are at a greater risk of developing CVDs, than smokers with healthier diets [9, 11]. However, previous studies mainly focused on individual dietary components, instead of assessment of overall dietary pattern [12–14]. Current evidence suggests that the impact of the entire diet on health outcomes is believed to be greater than that of individual dietary constituents [15, 16]. The Diet Quality Index (DQI) is a well-suited tool to assess dietary patterns and link dietary habits with diseases [17, 18]. Adherence to the DQI has been associated with a lower risk of CVDs, hypertension, and metabolic syndrome [19–21].

According to our literature review, very limited data are available concerning the dietary quality of smokers and its association with the incidence of CVDs and all-cause mortality. Additionally, no study investigates the joint association between smoking and diet quality among smokers, particularly in the Middle East and North Africa (MENA) region. Notably, previous studies have limitations since they did not consider the duration and intensity of smoking [3, 13, 22].

Considering the limited available data, this study aims to evaluate whether a smoker’s higher diet quality, either by itself or in combination with lower smoking intensity and duration or quitting smoking, can have an impact on their clinical outcomes. Hence, we examined the joint association between smoking intensity, smoking duration, and diet quality concerning the incidence of CVDs and all-cause mortality.

## Methods

### Study population

The Tehran lipid and glucose study (TLGS) is an ongoing population-based study aimed at identifying noncommunicable disease risk factors and promoting better lifestyles, with six follow-up examinations completed since 1999. The TLGS cohort consists of 15,005 participants aged 3 or more who undergo standardized physical exams, laboratory tests, and medical history updates every 3 years [23].

Since the collection of dietary data started from third examination, participants with complete dietary data on the third examination of TLGS and the new entries participants in the fourth examination were considered as baseline examinations and were followed until the end of sixth examination. In the third survey of the TLGS (2006–08), of 12 523 participants, 3686 were randomly selected for dietary assessment, and in the fourth survey (2009–2011), 7956 randomly selected subjects, agreed to complete dietary assessment. If a participant in the third examination had underreported or overreported energy intake (lower than 800 kcal/d and higher than 4200 kcal/d, respectively) (*n* = 233), was excluded but if their energy intake was in the normal range in the fourth examination (*n* = 100), their dietary intake in the fourth examination was included as their baseline dietary data. Finally, after excluding those with overunder reports of energy intake in the fourth examination (*n* = 502), 8914 participants with complete dietary data were included.

**Fig. 1:**
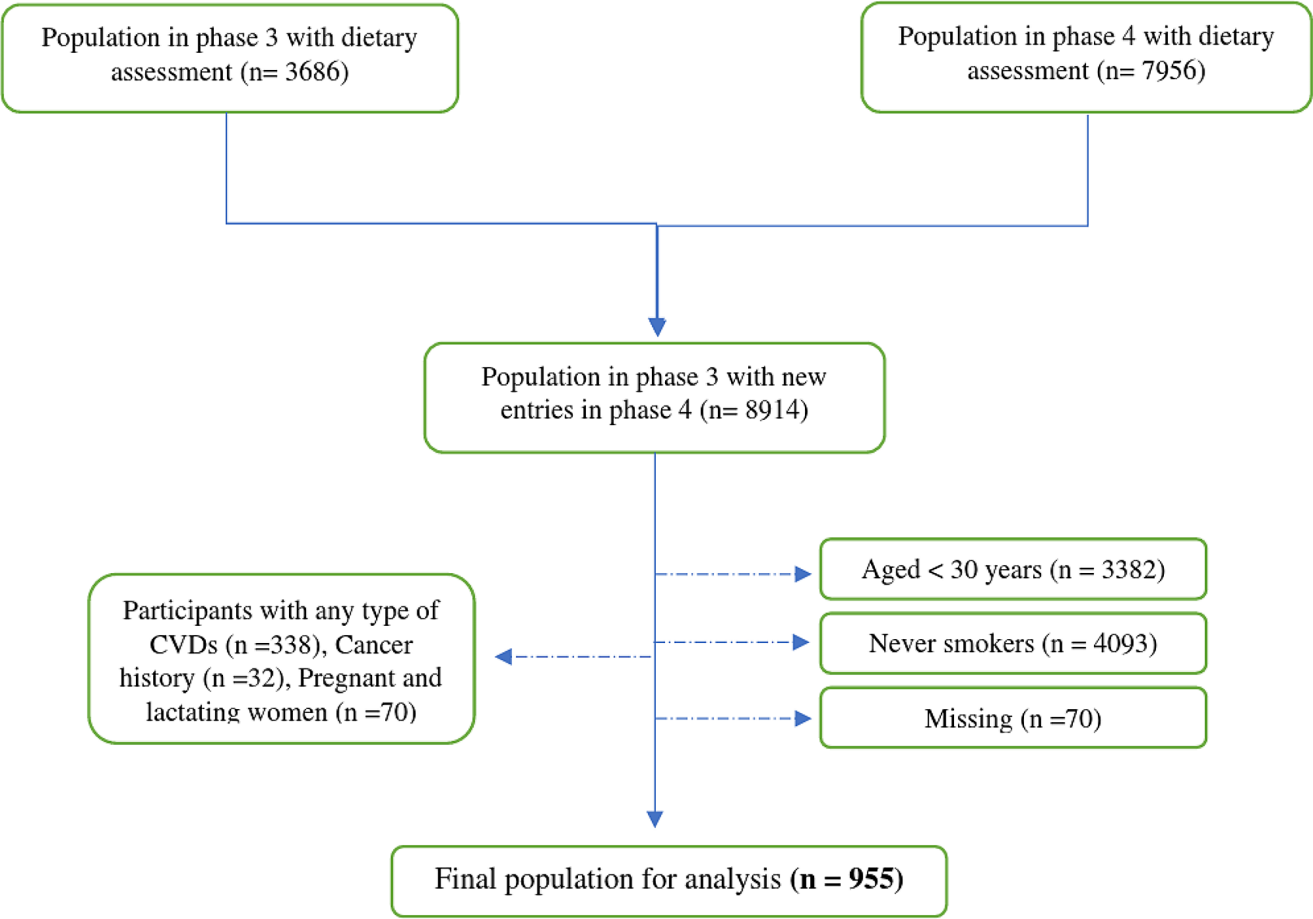
Diagram of the follow-up participants

For the present study, of 8914 individuals, participants aged < 30 years (*n* = 3382), never smokers (*n* = 4093) or participants with any type of CVDs (*n* = 338), cancer history (*n* = 32), pregnant and lactating women (*n* = 70) and missing data (*n* = 70) were excluded. Some of them may fall into more than one category. Finally, 955 smokers, free of CVDs entered into the study (Fig. 1) and were followed up until 20 March 2018. During the follow-up, all participants were assessed for any type of CVDs, and the mortality data were recorded.

### Dietary assessment

Skilled dieticians collected dietary data using a reliable and validated semiquantitative food frequency questionnaire (FFQ) comprising 168 items [24]. The study involved collecting information about the consumption frequency of various food items over the past year, categorized as daily, weekly, or monthly. The reported portion sizes of the consumed foods were converted from household measures to grams for analysis purposes. The energy and nutrient content of the food was calculated by utilizing the United States Department of Agriculture (USDA) food composition table (FCT) [25]. For local food items that were not listed in the USDA FCT, the Iranian FCT [26] was used.

### Demographic and clinical measurements

Trained interviewers conducted face-to-face interviews with participants using a standardized questionnaire to collect demographic variables. A telephone follow-up was performed annually to check for new medical events, with additional information gathered by a physician through a home visit or by accessing medical records if needed.

The validated Modifiable Activity Questionnaire (MAQ) was used to assess physical activity [27]. The MAQ questionnaire is divided into two categories based on recreational and occupational activities. Total physical activity was measured as metabolic equivalent minutes per week (MET-min/week) based on the frequency and duration of each activity over the preceding year.

Participants were asked to remain seated for 15 min before their blood pressure was measured. A qualified physician then took blood pressure readings twice, with at least a 30-second interval between measurements. A standard mercury sphygmomanometer was used for this purpose, which was calibrated by the Iranian Institute of Standards and Industrial Research. The mean of the two readings was recorded as the participant’s blood pressure. Height and weight were measured using a standard protocol. Participants were instructed to remove their shoes and wear light clothing. Height was measured using a stadiometer, while weight was measured using a calibrated weighing scale.

Serum glucose concentration was measured by drawing a blood sample between 7:00 and 9:00 a.m. after an overnight fast of 12–14 h. The laboratory kits used for the assay were supplied by Pars Azmon Inc. The assay employed an enzymatic colorimetric method with a glucose oxidase technique. The inter- and intra-assay variation coefficients for serum glucose concentration measurement are 2.2%, indicating a high level of accuracy and precision for the assay.

### Exposure definition

#### Current and former smokers

Current smokers were defined as participants who smoked either daily or occasionally [28]. Additionally, individuals who had quit smoking for less than a year were also considered current smokers [29]. Hence, participants who had ceased smoking for over a year were categorized as former smokers. Smoking intensity was measured based on the number of cigarettes smoked per day. To adjust for differences in the intensity and duration of smoking in study participants, the pack-year index was utilized. The pack-year was calculated by dividing the average number of cigarettes smoked per day by 20 and then multiplying it by the number of years of smoking [30].

#### Dietary quality index-international (DQI-I)

The dietary quality of participants was assessed using three dietary indices, including the DQI-I, Dietary Quality Index-Revised (DQI-R), and Mediterranean-Dietary Quality Index (Med.DQI). For DQI-I construction, we followed the method by Kim et al. [31]. In summary, the DQI-I is based on the North American Dietary Guidelines and emphasizes four primary elements: (1) variety in the intake of food groups including meat, poultry, fish, eggs, dairy, vegetables, fruits, grains, and beans, as well as variety within protein sources (0–20 points), (2) adequacy in the consumption of vegetables, fruits, grains, fiber, protein, and micronutrients like iron, calcium, and vitamin C (0–40 points), (3) moderation in the consumption of fat, saturated fat, cholesterol, sodium, and empty-calorie foods (0–30 points), and (4) overall balance in the ratio of macronutrients and fatty acids (0–10 points). The final DQI-I score ranges from 0 to 100, with a higher score indicating better diet quality.

#### Dietary quality index-revised (DQI-R)

The DQI-R is composed of ten components, four of which are identical to the original DQI (total fat, saturated fat, cholesterol, and calcium). The DQI-R now includes separate components for fruits, vegetables, grains, and iron, as well as new components for dietary moderation and diversity. Each component of the DQI-R is scored on a scale of 0 to 10, with a maximum score of 100 for the highest diet quality. Moderation in the diet pertains to the moderation of simple sugars, discretionary fat, sodium, and dietary alcohol consumption, while dietary diversity encompasses diversity in the intake of grains, fruits, vegetables, meat, and dairy products [32].

#### Mediterranean-dietary quality index (Med.DQI)

The Mediterranean diet has been linked to a reduced risk of chronic diseases, including all cancers, which makes the use of the Med-DQI important for assessing people’s nutritional status [33]. Med-DQI consists of seven components, including meat, fish, grains, fruits and vegetables, cholesterol, saturated fatty acids, and olive oil. Each component scored 0, 1, or 2 according to the recommended guidelines and assigned individuals a score between 0 and 14, with a higher score indicating a poor-quality diet.

### Outcome definition

CVDs were defined as a combined measure of coronary heart disease (CHD), stroke, or death due to cerebrovascular causes. Coronary heart disease-related events comprised cases of confirmed myocardial infarction (as determined by diagnostic electrocardiogram and biomarkers), possible myocardial infarction (established by positive electrocardiogram findings, symptoms or signs of a heart attack, and absence of biomarkers; or positive electrocardiogram findings and uncertain biomarkers), and CHD that was confirmed by angiography. The criteria for stroke were defined as a recently developed neurological impairment that persisted for a minimum of 24 h.

In the event of mortality, information was collected by an authorized local physician either from the hospital records or death certificates. The results obtained were assessed by an outcome committee that includes a chief researcher, an internist, an endocrinologist, a cardiologist, an epidemiologist, and the medical professional who compiles the outcome data.

### Statistical analyses

Statistical analysis was conducted using SPSS software (Statistical Package for the Social Sciences, version 26.0, SPSS Inc., Chicago, IL, USA). The primary characteristics of the total population, consisting of current and former smokers, are presented as percentages for categorical variables and mean ± standard deviation (SD) for quantitative variables. To compare the means of quantitative and categorical variables between the two groups of current and former smokers, an independent sample t-test, and chi-square analysis were used, respectively.

We utilized a Cox proportional hazard regression to determine the hazard ratio (HR) and 95% confidence interval (CI) for smoking intensity (measured by the number of cigarettes per day), smoking duration, and intensity (measured by pack years), and DQIs with regards to incidences of both CVDs and all-cause mortality.

Heavy smokers and light smokers as well as good and poor diet quality were determined based on the median intensity and duration of smoking and the median of DQIs, respectively. We divided the population into four groups: (1) heavy smokers with poor diet quality (reference group), (2) heavy smokers with good diet quality, (3) light smokers with poor diet quality, and (4) light smokers with good diet quality.

In addition, we measured the joint association between DQIs and smoking status in four groups: (1) current smokers with poor diet quality (reference group), (2) current smokers with good diet quality, (3) former smokers with poor diet quality, and (4) former smokers with good diet quality. Model 1 was adjusted for the variables that had significant associations with CVDs in the univariate analysis, including age, systolic blood pressure, fasting serum glucose, and job status (*P* < 0.05). Despite insignificance in univariate association, due to consistency with previous studies, Model 2 additionally adjusted for body mass index, physical activity, energy intake, marriage status, and education level. For each variable, the HR, and 95% CI, were reported. The time of follow-up was computed from the date of enrollment in the study until the first occurrence of CVDs events or the last follow-up date. All *P* values were based on two-sided tests and *P* values *<* 0.05 were considered significant.

## Results

Table 1 displays the baseline characteristics of the study population. The participants (88.60% men) had a mean age of 47.71 ± 10.61 years. The study had a mean follow-up period of 8.40 years for CVDs incidence and 8.80 years for all-cause mortality. The CVDs incidence and all-cause mortality rate in the current smoker group were 8.50% and 4.10%, respectively. Former smokers had a higher age, were more likely to be men and married, had higher fasting blood sugar and systolic blood pressure, and had a lower physical activity and employment percentage than current smokers. The incidence of CVDs in former smokers (12.60%) was higher than that in current smokers, but their overall mortality rate was similar to that of current smokers. Based on the DQI-I, DQI-R, and Med.DQI scores, the diet quality of former smokers was better than that of the current smokers group. The mean ± SD for the number of cigarettes smoked per day and pack-year index in the current smokers were equal to 8.89 ± 8.39 and 11.89 ± 12.15, respectively.

**Table 1:** Baseline characteristics of the studied population from Tehran lipid and glucose study*

	Total population (*n* = 955)	Current smokers (*n* = 638)	Former smokers (*n* = 317)	*P*-value**
Age (year)	47.71 ± 10.61	45.65 ± 9.87	51.86 ± 10.83	**0.001**
Male (%)	88.60	86.20	93.40	**0.001**
Body mass index (kg/m^2^)	27.23 ± 4.32	27.10 ± 4.49	27.49 ± 3.96	0.171
Physical activity (MET/min/wk)	2679 ± 4410	2916 ± 4810	2197 ± 3418	**0.009**
Education level (higher than diploma, %)	22.90	22.40	23.70	0.380
Job status (employed, %)	80.80	85.40	71.40	**0.001**
Marital status (married, %)	90.60	88.90	94.00	**0.011**
Fasting blood sugar (mg/dl)	99.73 ± 29.97	97.72 ± 27.62	103.78 ± 33.90	**0.006**
Systolic blood pressure (mmHg)	116.09 ± 16.52	113.97 ± 15.95	120.36 ± 16.84	**0.001**
DQI-I	63.09 ± 8.08	62.16 ± 8.12	64.97 ± 7.68	**0.001**
*Variety*	16.25 ± 2.97	16.22 ± 3.04	16.31 ± 2.82	0.675
*Adequacy*	32.08 ± 3.53	31.90 ± 3.67	32.46 ± 3.22	**0.016**
*Moderation*	12.33 ± 5.73	11.81 ± 5.79	13.38 ± 5.46	**0.001**
*Overall balance*	2.40 ± 2.50	2.22 ± 2.24	2.78 ± 2.24	**0.001**
DQI-R	70.71 ± 12.38	69.23 ± 12.49	73.67 ± 11.63	**0.001**
*Dietary diversity*	6.16 ± 1.32	6.15 ± 1.35	6.19 ± 1.25	0.697
*Dietary moderation*	6.21 ± 1.25	6.10 ± 1.29	6.42 ± 1.12	**0.001**
Med-DQI	6.05 ± 1.76	6.22 ± 1.77	5.69 ± 1.68	**0.001**
CVDs incidence (%)	9.80	8.50	12.60	**0.042**
All-cause mortality (%)	4.20	4.10	4.40	0.804

Abbreviations: DQI-I: Diet quality index-international; DQI-R: Diet quality index-revised; Med-DQI: Mediterranean-diet quality index; CVDs: Cardiovascular diseases.

* Significant differences are bolded.

**P-values were calculated for the differences between current and former smokers using chi-square and independent sample t-test.

Table 2 shows that in the fully-adjusted model (model 2), those in tertile 3 of smoking intensity (HR = 2.96; 95% CI = 1.48, 5.91) and pack-year index (HR = 4.41; 95% CI = 1.61, 12.08), had a higher risk for CVDs incidence than those in tertile 1. Additionally, the highest tertile of smoking intensity (HR = 8.28; 95% CI = 2.18, 31.42) and pack-year index (HR = 4.06; 95% CI = 1.01, 16.28) were associated with a higher risk of all-cause mortality in the fully adjusted model, than the lowest tertile. There was no significant relationship between the incidence of CVDs or all-cause mortality and the DQI-I index. In the crude and adjusted models, the second tertile of the DQI-R showed a lower risk for CVDs incidence compared to the first tertile (HR for adjusted model 2 = 0.43; 95% CI: 0.19, 0.96). Furthermore, the risk of all-cause mortality was higher in the third tertile of the Med-DQI index in both adjusted models, compared to the first tertile.

**Table 2:** The Hazard ratio and 95% CI for the incidence of CVDs and all-cause mortality based on the tertiles of diet quality indices and cigarette smoking intensity and duration among current smokers of the Tehran Lipid and Glucose Study†

	Cardiovascular diseases, HR (95%CI)			P-trend	All-cause mortality, HR (95%CI)			P-trend
	T1	T2	T3		T1	T2	T3	
**Smoking intensity (cigarettes/day)**								
Follow-up time (year)	8.6	8.6	7.9		9	8.8	8.6	
Person/time	1827	1982	1552		1911	2026	1666	
Incident case/Total	14/213	12/229	28/193		3/213	7/229	16/193	
Crude model	Ref	0.86 (0.39, 1.89)	**2.47 (1.27, 4.81)**	**0.001**	Ref	1.93 (0.48, 7.74)	**6.19 (1.79, 21.39)**	**0.001**
Model 1*	Ref	0.87 (0.39, 1.94)	**2.71 (1.37, 5.37)**	**0.001**	Ref	1.99 (0.47, 8.31)	**7.93 (2.19, 28.66)**	**0.001**
Model 2**	Ref	0.90 (0.40, 2.02)	**2.96 (1.48, 5.91)**	**0.001**	Ref	2.10 (0.49, 9.05)	**8.28 (2.18, 31.42)**	**0.001**
**Smoking intensity and duration (Pack. year)**								
Follow-up time (year)	8.8	8.5	7.9		8.9	8.9	8.6	
Person/time	1616	1597	1449		1644	1663	1560	
Incident case/Total	6/183	12/186	29/180		3/183	5/186	17/180	
Crude model	Ref	2.37 (0.83, 6.75)	**6.26 (2.41, 16.28)**	**0.001**	Ref	1.29 (0.29, 5.79)	**5.93 (1.73, 20.37)**	**0.001**
Model 1	Ref	1.80 (0.62, 5.24)	**4.03 (1.49, 10.87)**	**0.001**	Ref	0.98 (0.20, 4.62)	**4.43 (1.15, 17.03)**	**0.002**
Model 2**	Ref	1.82 (0.62, 5.27)	**4.41 (1.61, 12.08)**	**0.001**	Ref	0.96 (0.20, 4.65)	**4.06 (1.01, 16.28)**	**0.007**
**DQI-I**								
Follow-up time (year)	8.4	8.5	8.2		8.8	8.8	8.8	
Person/time	1808	1816	1761		1878	1875	1875	
Incident case/Total	14/213	18/213	22/212		7/213	11/213	8/212	
Crude model	Ref	1.23 (0.61, 2.47)	1.32 (0.66, 2.63)	0.441	Ref	1.36 (0.52, 3.59)	0.95 (0.33, 2.72)	0.897
Model 1*	Ref	1.16 (0.57, 2.38)	1.23 (0.61, 2.48)	0.555	Ref	1.16 (0.43, 3.15)	0.78 (0.27, 2.25)	0.607
Model 2**	Ref	1.13 (0.55, 2.32)	1.15 (0.56, 2.36)	0.711	Ref	1.22 (0.44, 3.38)	0.90 (0.30, 2.68)	0.814
**DQI-R**								
Follow-up time (year)	8.5	8.6	8.00		9	8.8	8.5	
Person/time	2308	1713	1364		2419	1756	1453	
Incident case/Total	26/268	10/200	18/170		12/268	8/200	6/170	
Crude model	Ref	**0.45 (0.21, 0.96)**	0.94 (0.50, 1.76)	0.497	Ref	0.78 (0.31, 1.99)	0.63 (0.22, 1.81)	0.379
Model 1*	Ref	**0.45 (0.20, 0.98)**	0.95 (0.51, 1.78)	0.542	Ref	0.59 (0.22, 1.56)	0.56 (0.19, 1.62)	0.223
Model 2**	Ref	**0.43 (0.19, 0.96)**	0.84 (0.43, 1.66)	0.359	Ref	0.55 (0.20, 1.52)	0.54 (0.17, 1.67)	0.220
**Med.DQI**								
Follow-up time (year)	8.1	8.4	8.7		8.6	8.9	8.9	
Person/time	1830	2253	1302		1945	2353	1330	
Incident case/Total	23/226	24/264	7/148		7/226	11/264	8/148	
Crude model	Ref	0.93 (0.51, 1.68)	0.51 (0.21, 1.21)	0.127	Ref	1.09 (0.40, 2.95)	1.81 (0.65, 4.49)	0.222
Model 1*	Ref	1.08 (0.59, 1.97)	0.62 (0.26, 1.47)	0.289	Ref	1.59 (0.58, 4.37)	**3.42 (1.15, 10.21)**	**0.023**
Model 2**	Ref	1.09 (0.59, 2.04)	0.62 (0.26, 1.50)	0.300	Ref	1.56 (0.55, 4.43)	**3.45 (1.12, 10.57)**	**0.025**

Abbreviations: DQI-I: Diet quality index-international; DQI-R: Diet quality index-revised; Med-DQI: Mediterranean-diet quality index.

*Adjusted for age, systolic blood pressure, fasting blood sugar, and job status.

**Additionally, adjusted for body mass index, physical activity, calorie intake, marriage status, and education level.

† Significant relations are bolded.

The joint association between smoking and diet quality (Table 3) was assessed on two levels: smoking intensity (cigarettes per day) and smoking intensity and duration (pack-year). Concerning smoking intensity, light smokers with good diet quality in comparison with heavy smokers with poor diet quality (reference group), had a lower risk of CVDs incidence in the fully multivariate-adjusted model according to DQI-I (HR = 0.42; 95% CI: 0.18, 0.99) and DQI-R (HR = 0.35; 95% CI: 0.15, 0.83). Additionally, only light smokers with good diet quality showed a lower risk for all-cause mortality compared to the reference group based on DQI-I (HR = 0.20; 95% CI: 0.05, 0.77) and DQI-R (HR = 0.19; 95% CI: 0.03, 0.98) in the fully multivariable-adjusted model. In addition, there was no significant joint association between Med-DQI and smoking intensity with CVDs incidence and all-cause mortality. Concerning the pack-year index, light smokers with a good DQI-R had a lower risk of CVDs than heavy smokers with poor diet quality in crude and adjusted models. (HR for adjusted model 2 = 0.32; 95% CI: 0.12, 0.83) Additionally, light smokers with good diet quality had a lower risk of all-cause mortality in crude and adjusted models according to the DQI-I (HR for adjusted model 2 = 0.08; 95% CI: 0.01, 0.69) and Med.DQI (HR for adjusted model 2 = 0.07; 95% CI: 0.00, 0.81).

**Table 3:** The Hazard ratio and 95% CI for the incidence of CVDs and mortality based on the diet quality indices joint association with smoking intensity and duration†

	Hazard Ratio (95%CI)			
	Heavy smoker-poor diet quality	Heavy smoker-good diet quality	Light smoker-poor diet quality	Light smoker-good diet quality
**Smoking intensity**				
**CVDs**				
***DQI-I***				
Crude model	Ref	1.34 (0.67, 2.65)	0.56 (0.25, 1.28)	0.47 (0.20, 1.06)
Model 1*	Ref	1.39 (0.70, 2.77)	0.62 (0.27, 1.41)	0.46 (0.20, 1.07)
Model 2**	Ref	1.32 (0.66, 2.66)	0.62 (0.27, 1.41)	**0.42 (0.18, 0.99)**
***DQI-R***				
Crude model	Ref	1.08 (0.53, 2.17)	0.52 (0.24, 1.11)	**0.40 (0.17, 0.92)**
Model 1*	Ref	1.05 (0.52, 2.14)	0.53 (0.25, 1.15)	**0.39 (0.17, 0.90)**
Model 2**	Ref	0.99 (0.48, 2.05)	0.53 (0.24, 1.15)	**0.35 (0.15, 0.83)**
***MED-DQI***				
Crude model	Ref	2.03 (0.97, 4.28)	0.68 (0.26, 1.79)	0.70 (0.29, 1.65)
Model 1*	Ref	1.91 (0.90, 4.06)	0.92 (0.34, 2.45)	0.58 (0.24, 1.40)
Model 2**	Ref	1.87 (0.88, 3.99)	0.91 (0.34, 2.43)	0.56 (0.23, 1.35)
**All-cause mortality**				
***DQI-I***				
Crude model	Ref	0.74 (0.27, 2.06)	0.50 (0.17, 1.47)	**0.25 (0.06, 0.91)**
Model 1*	Ref	0.62 (0.22, 1.75)	0.48 (0.16, 1.43)	**0.19 (0.05, 0.74)**
Model 2**	Ref	0.68 (0.24, 1.93)	0.49 (0.16, 1.50)	**0.20 (0.05, 0.77)**
***DQI-R***				
Crude model	Ref	1.67 (0.62, 4.46)	0.78 (0.27, 2.26)	0.25 (0.05, 1.20)
Model 1*	Ref	1.60 (0.58, 4.43)	0.83 (0.28, 2.43)	0.21 (0.04, 1.02)
Model 2**	Ref	1.62 (0.58, 4.52)	0.85 (0.28, 2.54)	**0.19 (0.03, 0.98)**
***MED-DQI***				
Crude model	Ref	1.89 (0.65, 5.45)	0.77 (0.20, 2.89)	0.50 (0.13, 1.87)
Model 1*	Ref	1.31 (0.44, 3.92)	0.96 (0.25, 3.64)	0.30 (0.07, 1.18)
Model 2**	Ref	1.40 (0.46, 4.24)	1.03 (0.27, 3.91)	0.29 (0.07, 1.21)
**Smoking duration and intensity**				
**Cardiovascular diseases**				
***DQI-I***				
Crude model	Ref	0.93 (0.46, 1.87)	**0.29 (0.11, 0.80)**	**0.35 (0.14, 0.85)**
Model 1*	Ref	0.94 (0.46, 1.90)	0.46 (0.17, 1.27)	0.46 (0.19, 1.11)
Model 2**	Ref	0.90 (0.44, 1.84)	0.44 (0.16, 1.23)	0.41 (0.16, 1.03)
***DQI-R***				
Crude model	Ref	0.71 (0.34, 1.47)	**0.28 (0.11, 0.70)**	**0.31 (0.12, 0.77)**
Model 1*	Ref	0.45 (0.18, 1.15)	0.45 (0.18, 1.15)	**0.38 (0.15, 0.96)**
Model 2**	Ref	0.64 (0.30, 1.35)	0.43 (0.17, 1.10)	**0.32 (0.12, 0.83)**
***MED-DQI***				
Crude model	Ref	1.36 (0.66, 2.79)	**0.24 (0.07, 0.87)**	0.52 (0.22, 1.23)
Model 1*	Ref	1.27 (0.62, 2.62)	0.44 (0.12, 1.61)	0.59 (0.24, 1.42)
Model 2**	Ref	1.25 (0.60, 2.59)	0.43 (0.11, 1.56)	0.55 (0.22, 1.37)
**All-cause mortality**				
***DQI-I***				
Crude model	Ref	0.70 (0.27, 1.79)	**0.27 (0.07, 0.96)**	**0.07 (0.01, 0.59)**
Model 1*	Ref	0.61 (0.23, 1.57)	0.41 (0.11, 1.53)	**0.08 (0.01, 0.65)**
Model 2**	Ref	0.66 (0.25, 1.71)	0.44 (0.11, 1.75)	**0.08 (0.01, 0.69)**
***DQI-R***				
Crude model	Ref	1.36 (0.56, 3.28)	0.42 (0.13, 1.35)	**0.11 (0.01, 0.89)**
Model 1*	Ref	1.32 (0.54, 3.22)	0.75 (0.22, 2.51)	0.12 (0.01, 1.01)
Model 2**	Ref	1.35 (0.55, 3.34)	0.84 (0.24, 2.92)	0.11 (0.01, 1.02)
***MED-DQI***				
Crude model	Ref	1.75 (0.66, 4.61)	0.50 (0.12, 2.00)	**0.11 (0.01, 0.95)**
Model 1*	Ref	1.40 (0.52, 3.75)	1.10 (0.26, 4.61)	**0.08 (0.01, 0.81)**
Model 2**	Ref	1.42 (0.52, 3.90)	1.24 (0.29, 5.22)	**0.07 (0.00, 0.81)**

Abbreviations: DQI-I: Diet quality index-international; DQI-R: Diet quality index-revised; Med-DQI: Mediterranean-diet quality index

*Adjusted for age, systolic blood pressure, fasting blood sugar, and job status.

**Additionally, adjusted for body mass index, physical activity, calorie intake, marriage status, and education level.

† Significant HRs are bolded.

In Supplementary Table 1, the risk of CVDs incidence and all-cause mortality was reported in former smokers compared to current smokers. After controlling for the confounding effect of various variables, former smokers had a 61% lower risk of all-cause mortality compared to current smokers (HR = 0.39; 95% CI: 0.17, 0.90). The risk of CVDs incidence and all-cause mortality among former smokers was not significantly different from that of light smokers, but in a fully adjusted model, former smokers had a lower risk of CVDs incidence (HR = 0.53; 95% CI: 0.31, 0.89) and mortality (HR = 0.22; 95% CI: 0.08, 0.55) when compared to heavy smokers.

**Fig. 2:**
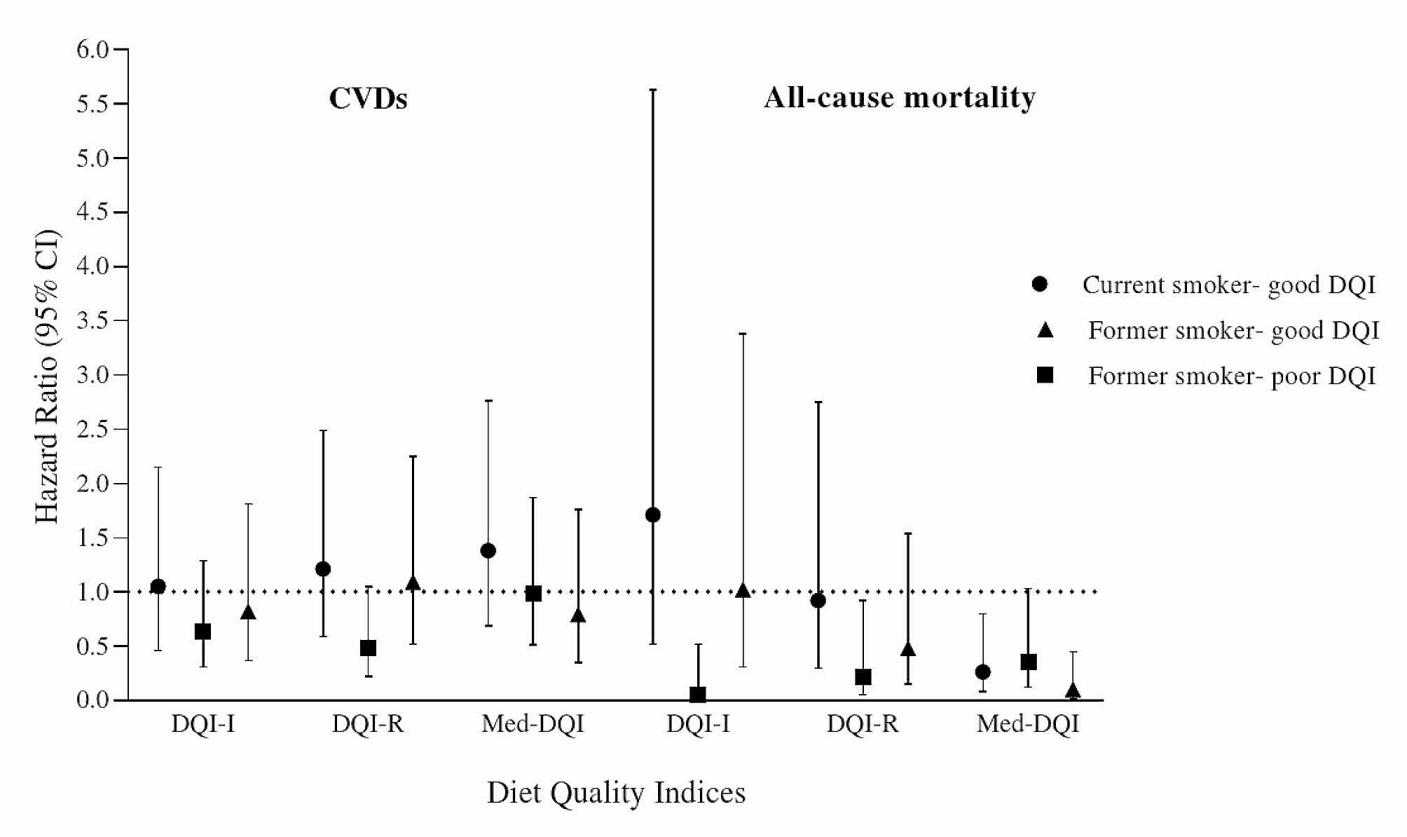
The Hazard ratio and 95% CI for incidence of CVDs and all-cause mortality based on the diet quality indices joint association with smoking status (Current smoker-poor DQI considered as the reference group)

Figure 2 and Supplementary Table 2, indicate the joint association between smoking and diet quality indices among current and former smokers. Former smokers with poor diet quality had a lower risk of all-cause mortality in adjusted models based on the DQI-I (HR = 0.05; 95% CI: 0.00, 0.52) and DQI-R (HR = 0.22; 95% CI: 0.05, 0.92) than current smokers with poor diet quality. According to the Med-DQI, former smokers who adhere to good diet quality, compared to current smokers with poor diet quality, had a lower risk for all-cause mortality in Model 2 (HR = 0.10; 95% CI: 0.02, 0.45). In addition, current smokers with good diet quality show a lower risk of mortality compared to current smokers with poor diet quality after accounting for potential covariates in the fully adjusted model (HR = 0.26; 95% CI: 0.08, 0.80).

## Discussion

We observed that cigarette smoking intensity and duration are independent contributors to CVDs incidence and all-cause mortality. The lower diet quality of current smokers, according to the Med-DQI, can increase the risk of mortality up to threefold. Light smokers with good diet quality had a lower risk of CVDs incidence and all-cause mortality compared to heavy smokers with poor diet quality. Current smokers with good Med-DQI had a lower risk of all-cause mortality compared to those with poor diet quality. This lower risk was more pronounced for former smokers than for current smokers.

Our study’s findings align with previous research [34, 35], indicating that cigarette smoking is strongly linked with CVDs incidence and all-cause mortality. Cigarette smoke contains polyaromatic hydrocarbons and oxidant gases [36]. It can impact CVDs by affecting all stages of atherosclerosis, vascular function, insulin sensitivity, serum lipid profile, advanced glycation end products (AGEs) synthesis, and gene methylation [35, 37, 38]. Also, according to our study, a low-quality diet among current smokers is linked to a higher risk of all-cause mortality. Studies that have measured the dietary quality of smokers using the DQI are scarce, but some studies have measured diet quality in the general population. The results from the Korean National Health and Nutrition Examination Surveys indicated that individuals with CVDs tend to have poorer diet quality than those without CVDs, as measured by DQI-I [39]. Asghari et al., have found a direct association between higher DQI-I scores and serum HDL-C levels [40]. However, a cross-sectional study by Daneshzad et al. did not find any significant association between DQI-I score and CVDs risk factors in type 2 diabetic women, possibly due to low statistical power [19].

According to our findings light smokers with a good diet quality had better outcomes than heavy smokers with a poor diet quality. To the best of our knowledge, no study has examined the joint association between smoking intensity, duration, and diet quality and the incidence of CVDs and all-cause mortality. Nonetheless, some studies examined the relationship between certain dietary constituents with clinical outcomes among smokers. According to Clark et al., dietary fiber intake can reduce the negative effects of second-hand smoke on mortality caused by coronary artery diseases [12]. Geng et al. also observed that individuals who are genetically predisposed to smoking and have lower DASH (dietary approach to stop hypertension) scores are at a higher risk of CVDs mortality [4]. Additionally, our study findings indicate that current smokers who maintain good diet quality have a lower risk of all-cause mortality as compared to current smokers with poor diet quality. Dauchet and colleagues have suggested that male smokers who consume more fruits and vegetables may have a lower risk of CVDs [14]. A review conducted by Vardavas et al. indicated that the health effects of smoking, including CVDs, and adherence to the Mediterranean diet, as a high-quality diet, may partially interact over time [38].

In general, smokers are more likely to experience negative clinical outcomes, which may be partially attributable to their poor nutritional status [9, 41]. Studies suggest that smokers are more susceptible to oxidative damage as a result of insufficient nutrient intake and the impact of smoking on nutrient metabolism [42]. Furthermore, smokers are more likely to consume excessive amounts of sodium [43], suffer from a decreased sense of taste [10], and suppressed appetite [44]. Smokers often tend to opt for less nutritious food options and consume less fiber, carotene, vitamin C, iron, and polyunsaturated fatty acids (PUFA) [42]. Notably, smoking can significantly affect the interaction between omega-3 and omega-6 PUFAs, and alter their metabolism [38]. Smokers have been shown to have lower adherence to high-quality diets like the Mediterranean diet [45]. Additionally, adherence to the Mediterranean diet has been linked to smoking cessation [46]. A study conducted by Roswall et al. found that adhering to a healthy Nordic food index had beneficial effects on CVDs risk only among former smokers [47]. They suggest that smoking cessation is associated with dietary changes that promote a healthier lifestyle, including an increase in fruit and vegetable consumption [47].

Several mechanisms are responsible for the observed joint association between smoking and diet quality. It has been observed that smokers had a lower level of antioxidants in their blood circulation [14]. However, the intake of fruits and vegetables with a high content of antioxidants was effective in controlling smoking-related oxidative damage [14]. Furthermore, Smoking’s negative effects on blood viscosity can be mitigated by flavonoids, which have been shown to inhibit platelet aggregation [14]. The fruit and vegetable intake has been linked to lower levels of C-reactive protein. Lower concentrations of C-reactive protein may reduce the negative effects of smoking on atherosclerosis and related mortality [14, 48]. In addition, a high-quality diet like the DASH diet has been shown to reduce oxidative stress and endothelial dysfunction through the lowering of dietary acid load [4]. Furthermore, adhering to a healthy diet may mitigate the deleterious effects of smoking on CVDs by promoting healthy gut microbiota [4]. The relationship between diet and smoking-associated mortality indicates that diet may serve as an indirect preventative measure by mitigating risk factors such as obesity and type 2 diabetes [4]. The Mediterranean diet, in particular, offers numerous health benefits like maintaining the ideal omega-6/omega-3 fatty acid ratio, modifying cell membrane composition and function, and gene expression [38]. Additionally, the Mediterranean diet can lower circulating low and very low-density lipoprotein cholesterol (LDL-C and VLDL-C) levels while increasing beneficial HDL-C [38].

However, we did not observe a significantly lower risk for CVDs incidence among heavy smokers with good diet quality compared to heavy smokers with poor diet quality. Millen et al. reported that smokers who adopt a heart-healthy diet are still at a higher risk of CVDs and all-cause mortality compared to non-smokers who follow a heart-healthy diet [3]. Studies have found that smoking-generated free radicals can counteract the dietary antioxidant effect due to oxidation and exert a pro-oxidative effect [49]. Also, we did not find a significant decrease in the risk of CVDs among former smokers, regardless of their diet quality, compared to current smokers with poor diet quality. Smoking can affect the hypothalamus, which reduces appetite and increases the level of catecholamines. This results in weight loss due to increased peripheral tissue energy consumption. However, after quitting smoking, with the elimination of nicotine’s appetite suppression effect, individuals may consume more food and experience weight gain. This weight gain, combined with other unknown confounding variables, could be the reason for the lack of statistical significance [44].

This study has several strengths, such as its novelty in the topic, prolonged follow-up period, focused research on the combined impact of smoking and diet quality, use of validated and reproducible FFQ to collect dietary data, and control for various potentially confounding variables. However, this study has some limitations that need to be considered. First, the number of participants in the pack-year group decreased due to incomplete data related to smoking duration. Second, the smaller sample size in the pack-year group resulted in lower statistical power. Third, like all observational studies, this study may be prone to measurement bias. Fourth, the study did not confirm the level of smoking intensity using serum markers such as cotinine. Additionally, no information was provided about changes in smoking habits during the study intervals. Fifth, the DQI-R computation did not include alcohol consumption because of a lack of available data. Lastly, because this was an observational study, unknown residual confounding effects could not be eliminated.

## Conclusions

In conclusion, the results of this prospective cohort study enhance the existing knowledge that smoking intensity, duration, and poor diet quality are significant risk factors for the incidence of CVDs and all-cause mortality. Light and former smokers exhibited a lower risk of developing CVDs and mortality, while a high-quality diet further strengthened this protective effect. Although smoking cessation remains the optimal approach to avoiding the negative health consequences of smoking, adherence to a high-quality diet could confer additional support, which could have substantial implications for clinical outcomes and public health.

### Electronic supplementary material

Below is the link to the electronic supplementary material.

Supplementary Material 1

## Data Availability

The data that support the findings of this study are available from the corresponding author upon reasonable request.

## Abbreviations

AGEs: Advanced Glycation End Products
CHD: Coronary Heart Disease
CI: Confidence Interval
CVDs: Cardiovascular Diseases
DALYs: Disability-Adjusted Life Years
DASH: Dietary Approaches to Stop Hypertension
DQI-I: Diet Quality Index-International
DQI-R: Diet Quality Index-Revised
DQI: Diet Quality Index
FCT: Food Composition Table
FFQ: Food Frequency Questionnaire
HDL-C: High-density Lipoprotein Cholesterol
HR: Hazard Ratio
LDL-C: Low-density Lipoprotein Cholesterol
MAQ: Modifiable Activity Questionnaire
Med-DQI: Mediterranean-Diet Quality Index
MENA: Middle East and North Africa
MET-min/week: Metabolic Equivalent minutes per week
PUFA: Polyunsaturated Fatty Acid
SD: Standard Deviation
TLGS: Tehran Lipid and Glucose Study
USDA: United States Department of Agriculture
VLDL-C: Very Low-density Lipoprotein Cholesterol

## References

[1] Vogel B, Acevedo M, Appelman Y, Merz CNB, Chieffo A, Figtree GA (2021). The Lancet women and cardiovascular disease commission: reducing the global burden by 2030. Lancet.

[2] Li Y, Cao G-y, Jing W-z, Liu J, Liu M (2023). Global trends and regional differences in incidence and mortality of cardiovascular disease, 1990– 2019: findings from 2019 global burden of disease study. Eur J Prev Cardiol.

[3] Millen BE, Quatromoni PA, Nam BH, O’Horo CE, Polak JF, Wolf PA (2004). Dietary patterns, smoking, and subclinical heart disease in women: opportunities for primary prevention from the Framingham Nutrition studies. J Am Diet Assoc.

[4] Geng T, Chang X, Wang L, Liu G, Liu J, Khor CC (2022). The association of genetic susceptibility to smoking with cardiovascular disease mortality and the benefits of adhering to a DASH diet: the Singapore Chinese Health Study. Am J Clin Nutr.

[5] Diseases GBo. 2023 https://www.healthdata.org/research-analysis/health-risks-issues/diet

[6] (GBD) GBoD. 2023 https://www.healthdata.org/research-analysis/health-risks-issues/smoking-and-tobacco

[7] Health effects of dietary risks (2019). In 195 countries, 1990–2017: a systematic analysis for the global burden of Disease Study 2017. Lancet.

[8] Mangoni AA, Sherwood RA, Swift CG, Jackson SH (2002). Folic acid enhances endothelial function and reduces blood pressure in smokers: a randomized controlled trial. J Intern Med.

[9] Dallongeville J, Marécaux N, Fruchart JC, Amouyel P (1998). Cigarette smoking is associated with unhealthy patterns of nutrient intake: a meta-analysis. J Nutr.

[10] Morabia A, Bernstein MS, Antonini S (2000). Smoking, dietary calcium and vitamin D deficiency in women: a population-based study. Eur J Clin Nutr.

[11] Stamler J, Rains-Clearman D, Lenz-Litzow K, Tillotson JL, Grandits GA (1997). Relation of smoking at baseline and during trial years 1–6 to food and nutrient intakes and weight in the special intervention and usual care groups in the multiple risk factor intervention trial. Am J Clin Nutr.

[12] Clark ML, Butler LM, Koh WP, Wang R, Yuan JM (2013). Dietary fiber intake modifies the association between secondhand smoke exposure and coronary heart disease mortality among Chinese non-smokers in Singapore. Nutrition.

[13] Hansen RD, Albieri V, Tjønneland A, Overvad K, Andersen KK, Raaschou–Nielsen O (2013). Effects of smoking and antioxidant micronutrients on risk of colorectal cancer. Clin Gastroenterol Hepatol.

[14] Dauchet L, Montaye M, Ruidavets JB, Arveiler D, Kee F, Bingham A (2010). Association between the frequency of fruit and vegetable consumption and cardiovascular disease in male smokers and non-smokers. Eur J Clin Nutr.

[15] Petersen KS, Kris-Etherton PM. Diet quality assessment and the relationship between diet quality and cardiovascular disease risk. Nutrients. 2021;13(12). 10.3390/nu13124305 10.3390/nu13124305 PMC8706326 34959857

[16] Willett W. Nutritional epidemiology. Oxford University Press; 2012.

[17] Gerber M (2006). Qualitative methods to evaluate Mediterranean diet in adults. Public Health Nutr.

[18] Williams J, Townsend N, Rayner M, Jayawardena R, Katulanda P, Manoharan S (2019). Diet quality of adolescents in rural Sri Lanka based on the Diet Quality Index-International: findings from the ‘Integrating Nutrition Promotion and Rural Development’ project. Public Health Nutr.

[19] Daneshzad E, Larijani B, Azadbakht L (2019). Diet quality indices and cardiovascular diseases risk factors among diabetic women. J Sci Food Agric.

[20] Payandeh N, Shahinfar H, Jafari A, Babaei N, Djafarian K, Shab-Bidar S (2021). Mediterranean diet quality index is associated with better cardiorespiratory fitness and reduced systolic blood pressure in adults: a cross-sectional study. Clin Nutr ESPEN.

[21] Abdurahman A, Bule M, Fallahyekt M, Abshirini M, Azadbakht L, Qorbani M (2021). Association of Diet Quality and Food Insecurity with metabolic syndrome in obese adults. Int J Prev Med.

[22] Li Z, Kesse-Guyot E, Dumas O, Garcia-Aymerich J, Leynaert B, Pison C (2017). Longitudinal study of diet quality and change in asthma symptoms in adults, according to smoking status. Br J Nutr.

[23] Azizi F, Zadeh-Vakili A, Takyar M (2018). Review of Rationale, Design, and initial findings: Tehran lipid and glucose study. Int J Endocrinol Metab.

[24] Esfahani FH, Asghari G, Mirmiran P, Azizi F (2010). Reproducibility and relative validity of food group intake in a food frequency questionnaire developed for the Tehran lipid and glucose study. J Epidemiol.

[25] Satija A, Hu FB (2012). Cardiovascular benefits of dietary fiber. Curr Atheroscler Rep.

[26] Azar M, Sarkisian E (1980). Food composition table of Iran: National Nutrition and food research institute.

[27] Momenan AA, Delshad M, Sarbazi N, Rezaei Ghaleh N, Ghanbarian A, Azizi F (2012). Reliability and validity of the modifiable activity questionnaire (MAQ) in an Iranian urban adult population. Arch Iran Med.

[28] Hadaegh F, Derakhshan A, Mozaffary A, Hasheminia M, Khalili D, Azizi F (2016). Twelve-Year Cardiovascular and Mortality Risk in Relation to Smoking habits in type 2 Diabetic and non-diabetic men: Tehran lipid and glucose study. PLoS ONE.

[29] Wang J, Bai Y, Zeng Z, Wang J, Wang P, Zhao Y (2022). Association between life-course cigarette smoking and metabolic syndrome: a discovery-replication strategy. Diabetol Metab Syndr.

[30] Leffondré K, Abrahamowicz M, Siemiatycki J, Rachet B (2002). Modeling smoking history: a comparison of different approaches. Am J Epidemiol.

[31] Kim S, Haines PS, Siega-Riz AM, Popkin BM (2003). The Diet Quality Index-International (DQI-I) provides an effective tool for cross-national comparison of diet quality as illustrated by China and the United States. J Nutr.

[32] Newby PK, Hu FB, Rimm EB, Smith-Warner SA, Feskanich D, Sampson L (2003). Reproducibility and validity of the Diet Quality Index revised as assessed by use of a food-frequency questionnaire. Am J Clin Nutr.

[33] Bondia-Pons I, Mayneris-Perxachs J, Serra-Majem L, Castellote AI, Mariné A, López-Sabater MC (2010). Diet quality of a population sample from coastal north-east Spain evaluated by a Mediterranean adaptation of the diet quality index (DQI). Public Health Nutr.

[34] Ding N, Sang Y, Chen J, Ballew SH, Kalbaugh CA, Salameh MJ (2019). Cigarette smoking, Smoking Cessation, and long-term risk of 3 major atherosclerotic diseases. J Am Coll Cardiol.

[35] Al-Delaimy WK, Manson JE, Solomon CG, Kawachi I, Stampfer MJ, Willett WC (2002). Smoking and risk of coronary heart disease among women with type 2 diabetes mellitus. Arch Intern Med.

[36] Ambrose JA, Barua RS (2004). The pathophysiology of cigarette smoking and cardiovascular disease: an update. J Am Coll Cardiol.

[37] Maas SCE, Mens MMJ, Kühnel B, van Meurs JBJ, Uitterlinden AG, Peters A (2020). Smoking-related changes in DNA methylation and gene expression are associated with cardio-metabolic traits. Clin Epigenetics.

[38] Vardavas CI, Flouris AD, Tsatsakis A, Kafatos AG, Saris WH (2011). Does adherence to the Mediterranean diet have a protective effect against active and passive smoking?. Public Health.

[39] Cho IY, Lee KM, Lee Y, Paek CM, Kim HJ, Kim JY, et al. Assessment of dietary habits using the diet quality index-international in cerebrovascular and cardiovascular disease patients. Nutrients. 2021;13(2). 10.3390/nu13020542 10.3390/nu13020542 PMC7914702 33562317

[40] Asghari G, Mirmiran P, Hosseni-Esfahani F, Nazeri P, Mehran M, Azizi F (2013). Dietary quality among tehranian adults in relation to lipid profile: findings from the Tehran lipid and glucose study. J Health Popul Nutr.

[41] Gariballa S, Forster S (2009). Effects of smoking on nutrition status and response to dietary supplements during acute illness. Nutr Clin Pract.

[42] Margetts BM, Jackson AA (1993). Interactions between people’s diet and their smoking habits: the dietary and nutritional survey of British adults. BMJ.

[43] Choi KH, Park MS, Kim JA, Lim JA (2015). Associations between Excessive Sodium Intake and Smoking and Alcohol Intake among Korean men: KNHANES V. Int J Environ Res Public Health.

[44] Zhu P, Pan XF, Sheng L, Chen H, Pan A (2017). Cigarette smoking, diabetes, and diabetes complications: call for urgent action. Curr Diab Rep.

[45] Gangadi M, Kalpourtzi N, Gavana M, Vantarakis A, Chlouverakis G, Hadjichristodoulou C (2021). Prevalence of tobacco smoking and association with other unhealthy lifestyle risk factors in the general population of Greece: results from the EMENO study. Tob Prev Cessat.

[46] Parekh TM, Wu C, McClure LA, Howard VJ, Cushman M, Malek AM (2019). Determinants of cigarette smoking status in a national cohort of black and white adult ever smokers in the USA: a cross-sectional analysis of the REGARDS study. BMJ Open.

[47] Roswall N, Sandin S, Scragg R, Löf M, Skeie G, Olsen A (2015). No association between adherence to the healthy nordic food index and cardiovascular disease amongst Swedish women: a cohort study. J Intern Med.

[48] Ahmadirad H, Teymoori F, Mokhtari E, Jahromi MK, Norouzzadeh M, Tavakkoli S (2023). Serum C-peptide level and the risk of cardiovascular diseases mortality and all-cause mortality: a meta-analysis and systematic review. Front Cardiovasc Med.

[49] Chaiter Y, Gruber SB, Ben-Amotz A, Almog R, Rennert HS, Fischler R (2009). Smoking attenuates the negative association between carotenoids consumption and colorectal cancer risk. Cancer Causes Control.

