# Supplementary material for: Cigarette smoking and cardiovascular disease incidence and all-cause mortality: the modifying role of diet quality
Source: BMC Public Health. 2024 Apr 12;24:1021. doi: 10.1186/s12889-024-18468-z (PMC11010434; doi:10.1186/s12889-024-18468-z)
Supplement: Supplementary file 1 — Supplementary Material 1 [file 12889_2024_18468_MOESM1_ESM.docx]

| **Supplementary Table 1.** The Hazard ratio and 95% CI for mortality and the incidence of CVD according to smoking status^*^. | | |
| --- | --- | --- |
|  | **Former vs current smoker, HR (95%CI)** | |
| **Cardiovascular disease** |  |  |
| Crude model | Ref | **1.43 (0.93, 2.20)** |
| Model 1* | Ref | 0.76 (0.47, 1.21) |
| Model 2** | Ref | 0.74 (0.46, 1.19) |
| **All-cause mortality** |  |  |
| Crude model | Ref | 0.84 (0.40, 1.77) |
| Model 1* | Ref | **0.31 (0.13, 0.70)** |
| Model 2** | Ref | **0.39 (0.17, 0.90)** |
|  | **Former vs light smokers, HR (95%CI)** | |
| **Cardiovascular disease** |  |  |
| Crude model | Ref | **2.34 (1.29, 4.22)** |
| Model 1* | Ref | 1.34 (0.71, 2.51) |
| Model 2** | Ref | 1.30 (0.69, 2.45) |
| **All-cause mortality** |  |  |
| Crude model | Ref | 1.50 (0.57, 3.94) |
| Model 1* | Ref | 0.64 (0.22, 1.83) |
| Model 2** | Ref | 0.92 (0.30, 2.87) |
|  | **Former vs heavy smokers, HR (95%CI)** | |
| **Cardiovascular disease** |  |  |
| Crude model | Ref | 1.00 (0.63, 1.60) |
| Model 1* | Ref | **0.55 (0.32, 0.92)** |
| Model 2** | Ref | **0.53 (0.31, 0.89)** |
| **All-cause mortality** |  |  |
| Crude model | Ref | 0.57 (0.26, 1.25) |
| Model 1* | Ref | **0.17 (0.07, 0.44)** |
| Model 2** | Ref | **0.22 (0.08, 0.55)** |
| * Significant HRs are bolded. | | |

| **Supplementary Table 2.** The Hazard ratio and 95% CI for CVDs incidence and all-cause mortality based on the diet quality indices joint association with smoking status^†^. | | | | |
| --- | --- | --- | --- | --- |
|  | **Hazard Ratio (95%CI)** | | | |
|  | Current smoker-poor DQI | Current smoker-good DQI | Former smoker-  poor DQI | Former-smoker-  good DQI |
| **Cardiovascular diseases** |  |  |  |  |
| ***DQI-I*** |  |  |  |  |
| Crude model | Ref | 1.14 (0.67, 1.95) | 1.27 (0.67, 2.40) | **1.97 (1.13, 3.41)** |
| Model 1* | Ref | 1.05 (0.61, 1.80) | 0.67 (0.34, 1.31) | 0.98 (0.54, 1.75) |
| Model 2** | Ref | 1.005 (0.46, 2.15) | 0.64 (0.31, 1.29) | 0.82 (0.37, 1.81) |
| ***DQI-R*** |  |  |  |  |
| Crude model | Ref | 1.27 (0.74, 2.17) | 1.01 (0.50, 2.06) | **2.33 (1.36, 3.98)** |
| Model 1* | Ref | 1.16 (0.68, 2.007) | 0.55 (0.26, 1.15) | 1.15 (0.65, 2.03) |
| Model 2** | Ref | 1.21 (0.59, 2.49) | 0.48 (0.22, 1.05) | 1.09 (0.52, 2.25) |
| ***MED-DQI*** |  |  |  |  |
| Crude model | Ref | 1.37 (0.80, 2.35) | **1.89 (1.04, 3.46)** | 1.72 (0.95, 3.09) |
| Model 1* | Ref | 1.32 (0.77, 2.27) | 1.11 (0.59, 2.07) | 0.82 (0.44, 1.53) |
| Model 2** | Ref | 1.38 (0.69, 2.76) | 0.98 (0.51, 1.87) | 0.79 (0.35, 1.76) |
| **All-cause mortality** |  |  |  |  |
| ***DQI-I*** |  |  |  |  |
| Crude model | Ref | 1.22 (0.56, 2.64) | 0.36 (0.08, 1.61) | 1.97 (0.88, 4.40) |
| Model 1* | Ref | 0.92 (0.42, 2.01) | **0.11 (0.02, 0.52)** | 0.65 (0.28, 1.50) |
| Model 2** | Ref | 1.71 (0.52, 5.63) | **0.05 (0.00, 0.52)** | 1.02 (0.31, 3.38) |
| ***DQI-R*** |  |  |  |  |
| Crude model | Ref | 1.07 (0.49, 2.31) | 0.71 (0.23, 2.19) | 1.45 (0.63, 3.31) |
| Model 1* | Ref | 0.77 (0.35, 1.69) | **0.23 (0.07, 0.77)** | 0.46 (0.19, 1.10) |
| Model 2** | Ref | 0.92 (0.30, 2.75) | **0.22 (0.05, 0.92)** | 0.48 (0.15, 1.54) |
| ***MED-DQI*** |  |  |  |  |
| Crude model | Ref | 0.48 (0.21, 1.10) | 0.90 (0.37, 2.17) | 0.74 (0.31, 1.78) |
| Model 1* | Ref | **0.41 (0.18, 0.96)** | **0.37 (0.15, 0.93)** | **0.22 (0.08, 0.60)** |
| Model 2** | Ref | **0.26 (0.08, 0.80)** | 0.35 (0.12, 1.03) | **0.10 (0.02, 0.45)** |
| **Abbreviations: DQI-I**, Diet quality index-international**;** **DQI-R**, Diet quality index-revised**;** **Med-DQI**, Mediterranean-diet quality index.  *Adjusted for age, systolic blood pressure, fasting blood sugar, and job status.  **Additionally adjusted for BMI, physical activity, calorie intake, marriage status, and education level.  † Significant HRs are bolded. | | | | |
